# A cluster randomized trial: Exploratory quantitative analysis comparing resident outcomes for two approaches to promote non‐pharmacological, resident‐centered dementia care in nursing homes

**DOI:** 10.1002/alz.083708

**Published:** 2025-01-09

**Authors:** Victoria Shier, Yuna H. Bae‐Shaaw, Neeraj Sood, Felicia M Chew, Catherine V Piersol, Cara A. Lekovitch, Dominique H. Como, Carin M. Wong, Mike Morris, Natalie E Leland

**Affiliations:** ^1^ University of Southern California, Los Angeles, CA USA; ^2^ Thomas Jefferson University, Philadelphia, PA USA; ^3^ University of Pittsburgh, Pittsburgh, PA USA; ^4^ Powerback Rehabilitation, kennett Square, PA USA

## Abstract

**Background:**

Resident advocates and national nursing home dementia care initiatives have prioritized non‐pharmacological approaches to manage behavioral and psychological symptoms of Alzheimer’s Disease and related dementias. Evidence supports both team‐ and problem‐based approaches to non‐pharmacological dementia care, but the comparative effectiveness of these two approaches has not been examined.

**Method:**

We implemented a cluster randomized controlled trial in 53 nursing homes ot compare the team‐based and problem‐based approaches to dementia care. Nursing homes in the team‐based approach arm completed a five‐module facility‐wide training, which provided a common language and shared knowledge across all staff. The problem‐based approach engaged in discipline‐specific web‐based training. Resident assessment data from the Minimum Data Set 3.0 was analyzed. Primary outcomes were antipsychotic medication use, behavioral symptoms, rejection of care, and wandering. Secondary outcomes were unintentional weight loss, falls, depression and restraint use. We compared change in outcomes from the six months baseline period to six months follow‐up period across study arms using a difference‐in‐differences model.

**Result:**

The percentage of residents with wandering increased 3.51 percentage points (p = 0.02) in the team‐based approach arm compared to the problem‐based approach arm, with no evidence of associated adverse events (e.g., falls, unintentional weight loss). There was no statistically significant difference in antipsychotic medication use or other resident outcomes across arms. However, the reduction in rejection of care and the increase in behavioral symptoms in the team‐based approach compared to the problem‐based approach were potentially clinically meaningful differences between the two arms.

**Conclusion:**

Results suggest a benefit for resident wandering in the team‐based approach. While findings are exploratory, training for all nursing home staff that accounts for diverse education and training needs may influence care delivery and have benefits for residents with dementia.

